# The Dentist and His Associates

**Published:** 1900-11

**Authors:** Charles Otis Kimball

**Affiliations:** New York City


### International Dental Journal.

Vol. XXI. November, 1900. No. 11

### Original Communications.[1]

### THE DENTIST AND HIS ASSOCIATES.[2]

BY CHARLES OTIS KIMBALL, A.B., M.D., NEW YORK CITY.

When, as Bryant says,

> "The melancholy days are come, the saddest of the year,
> Of wailing winds and naked woods and meadows brown and sere;
> Heaped in the hollows of the grove the autumn leaves lie dead,
> They rustle to the eddying gust and to the rabbit's tread,"

and we note the changing season, it is not strange if our thoughts take on a tinge of sadness as we remember that "we all do fade as a leaf." But there is another lesson drawn from the crimson leaf wavering down in the mellow air, that turns our thoughts from the vanishing past to the advancing future. For as each leaf falls reluctantly from the twig where it has hung the summer through to find its final rest, its life-work done, a tiny bud giving promise of a new year's verdure is revealed by the loosening stem.

Science says, "No life without a life," and looks backward to see whence it came. Faith says, "Every life for life," and looking

---

[1] The editor and publishers are not responsible for the views of authors of papers published in this department, nor for any claim to novelty, or otherwise, that may be made by them. No papers will be received for this department that have appeared in any other journal published in the country.

[2] Read before The New York Institute of Stomatology, June 5, 1900.

forward sees in the future the meaning and value of the present. It is by no mere chance that a mother gives her life for her child, it may be in one agony of surrender, or more often, minute by minute through long years, sacrificing her life for his, for under mother and child, leaf and bud, is Nature's fundamental law, that each individual prepares the way for and helps on his successor.

I am here to-night to speak of one phase of this great law. To plead for continuity of life, not merely physical, but that higher life of intelligent and persistent effort in following out an idea, which is the meaning of every profession.

We have lately listened to a helpful paper upon "The Successful Dentist," which showed how essential to true success is soundness of character and earnestness of purpose; how well-directed efforts stimulated by love for pre-eminence, desire for ampler compensation, and a sincere wish to help others, achieve a degree of knowledge, skill, and delicacy of touch, which combined with patience, gentleness, and sympathy for patients win a confidence firm and abiding.

My theme, symbolized by the hand passing a torch, is that one life finds its fullest development in transmitting life, only as we give do we live; so it carries the thought of the essayist one step farther. "The Successful Dentist," as he takes a thoughtful view of his life and its experiences, as he thinks of all he has received from the past, all he has acquired in the present by careful observation and patient self-forgetful work, using the faculties given to him, must feel that this fund of learning, observation, experience, and skill is a great talent intrusted to him by no accident, but of purpose (call it what you will, it seems to me divine), which should not only be used to the full, but in some way transmitted as a sacred trust to help and uplift other lives,—he must pass the torch,—and the question arises, How may such a professional life be best transmitted?

As the years go by each wise decision or skilful effort makes its deepening impression on the brain of the dentist himself, fitting him for still better work and more accurate judgment; besides, since he is not working upon mere matter, but upon observing and thinking creatures, who find that they are helped by his efforts, he is training their minds into a habit of confidence in him, which deepens and broadens with every such experience, depending rather upon the expression of his character than upon their accurate knowledge of what he is doing. This confidence becomes a part of his funded increment of life, his capital. How can he make use of it to enlarge his sphere of usefulness beyond the working power of one pair of hands?

By a subtle law of sympathy confidence is communicated from one to another, and so a practice grows till it fills his life full and he goes on rejoicing in his strength, like a skilled fencer, with conscious delight in each difficulty mastered. He is strong and elastic, still capable of his best work, but the years are telling upon him, and possibly a little something of his youthful enthusiasm has gone, though the work to be done is ever growing in amount, till the ascending curve of judgment and experience is crossed by the descending curve of physical strength, while the calls upon that judgment and experience are increasing,

> "Till at length the burden seems
> Greater than our strength can bear,
> Heavy as the weight of dreams
> Pressing on us everywhere."

How, then, can this situation be met as the work becomes too great for him?

Moreover, as his experience lengthens and his judgment matures he acquires a quickness of perception, a power of seeing through a difficult situation, a clearness in diagnosis, which becomes valuable. In a moment's time he can advise or suggest courses of action which may take hours or months for completion; his mental power has outgrown his physical powers, he can plan more than he can possibly execute alone, while his judgment becomes nearly unfailing. This is the crowning glory of a professional life, this makes a man a consultant, when his advice is sought not by patients merely but by his fellow-craftsmen. How can a man best use this experience and judgment to help his professional brethren?

Aside from these various considerations, most of which bear more or less upon his natural desire for his own gain, there is an unselfish side of the question. For there are many patients endeared to him by long friendship whose means will not allow them to pay his prices, yet his time is full at the higher rate. What a relief to him and what a comfort to them to have some one who can take up his work, carrying it on to a fuller perfection in complete sympathy with him and faithfulness to them; to whom he can intrust them, and while watching and advising, feel that he still holds them in his care even if they are not served by his own hands! How can he do this?

Allow me to repeat these questions that I have raised: 1. How can a dentist best transmit his professional life? 2. How can he utilize as part of his capital his patients' confidence? 3. How can he be relieved from the overpressure of a practice too large? 4. How can he use his acquired judgment and experience with the best result? 5. How can he best help his old patients when unable personally to serve them? One answer fits them all: by selecting, training, retaining, and advising an associate.

Let us consider this answer under four heads: 1. The associate, —how to choose him. 2. What are his needs? 3. What should characterize the relation between dentist and associate. 4. By what methods can this relation be sustained?

As I look around I see before me the men who have been in my mind as I have tried to picture the successful dentist in his duties as well as his needs, and if you will allow me I will change the person and speak directly to you as representing "The Successful Dentist" needing an associate.

### 1. THE ASSOCIATE.

A wise lawyer once said that next in importance to choosing a wife came the selection of a business partner. Hence in any thought of close association with another the most important thing to be considered is the man himself, the real man, his character, the mint-mark by which he is reckoned in the sum of life, for you are not merely teaching a student who may go away, but you are planning for a friend who shall be like a son to you, and who should fairly reflect every gift and grace you yourself may possess.

You must seek for truth, honesty, faithfulness, perseverance, courage, self-confidence, self-forgetfulness, a high sense of honor, and reverence for womanhood, these elements of character, like the facets of a cut gem, lead the eye to it and reveal its beauty. Next to these come his personal habits, for the dentist more than any other professional man is brought continually into the closest intimate personal contact with his patients. Hence he should have cleanness of thought and speech, chasteness of life, neatness of person and clothing, sweetness of breath, always suggesting physical and moral purity. And then giving vitality to all these, making them effective, note his purpose in life,—is it deep and true? has he lighted that lamp of sacrifice which alone can show the way along the path of duty? is he ready to devote his life faithfully to his profession? Such men there are,—they are worth seeking long for.

Nor are these the only things to be considered; you must seek for a good appearance, ease of manner and address, courteous and affable ways, obligingness, punctuality, promptness, for these will all tell in his work. Then as to his ability or rather adaptability to the special work before him, is he handy with tools, accustomed to nicety of execution? can he see straight and work to a finish? And you may well ask, What are his amusements and interests outside of his work? What has been his general education, is it such as barely suffices to pass his Regents' examinations, or such as shall put him on a par with the doctors and lawyers and ministers of the community,—viz., a good college education? can he think and speak clearly and intelligently? is his language good, so that he is at home with the best-educated men and women? Lastly, how careful has been his professional training? You must seek to have his preparation as thorough or more so than your own. You must bear in mind that his life is to be the standard of the profession in days to come, therefore must see to it that he lays a broad and deep foundation. He should have studied medicine as a basis on which to build his special study and training as a dentist, in this way your personal influence will directly tend towards raising the general standard of the profession.

### 2. HIS NEEDS.

Let us now consider what such a young man just graduating from a dental school needs upon his entry into active professional life. In the first place, he needs opportunity for constant practice in operating under your eye and direction till the most delicate and difficult operations are certain in his hands; this practice he cannot get in any dental school of which I am aware. They must teach theory with some practice; your office should be a post-graduate school where he will reduce his theories to perfect practice under your trained and watchful eye. Every operation should be criticised kindly and fairly but unsparingly till his standard of perfection in work comes up to your own, and this repeated, showing him the reasons, till his own standard of criticism is as high as yours.

Then in like way he needs criticism upon his judgments in order to train his judgment. Judgment is based largely upon one's own experience, to a less degree upon that of others. He is young, has had little or no experience upon which to base a sound judgment. Your long experience should be at his service, recalling observations of similar cases, pointing out the far-away results of operations, calling attention to minute diagnostic signs, and so putting him in the way of making wise decisions. These should be referred to you for confirmation, till gradually he learns to decide just as clearly and accurately as you do. Thus his judgment is trained to wise and independent action. Meanwhile, it is backed up by the weight of your authority strengthening his patients' confidence in him. In the simple cases of every-day practice he soon learns to decide wisely and act promptly, but even this responsibility is assumed gradually, while in the doubtful and puzzling cases where he is not quite sure of himself, he leans upon you as a rock, till time and experience fit him in turn to be a supporter to others. In these difficult cases every young practitioner needs a chance for ready consultation. His patient is in the chair, something must be done immediately, and many a time (especially at the first) your suggestion will come to him as a great relief, not only giving him an outline to work to, but perhaps putting the clue to the whole situation in his hands. He needs also your encouragement in study and research, in thinking for himself that he may grow in wisdom. A hint dropped now and then, a leading question, a problem given for solution, these will stimulate his mind and lead to a fuller development of all his powers.

### 3. THE RELATION.

Now, what shall be the nature of the relation between you and your associate, chosen so carefully and needing so much? First, it must be *as just to him* as to you. His life-work is before him, and the growth of a personal independent practice which shall be his own must be clearly contemplated from the start. Much of his practice will come by the inevitable growth pertaining to faithful work of which we have already spoken, and only a small part will be received directly from you. But if he is such a man as he should be, and trained as you should train him, you should take pains to see that he is introduced in your best families, till he is known as one whom you trust and upon whom they may safely rely. The relation must be cordial and friendly; he must feel that you are interested in his success, and indeed that you are in full sympathy with all his life. Your advice in other things than mere professional matters will be sought for and valued, and so your own personality will be impressed upon him, and his gold will bear the stamp of your character.

Second, it must also be a relation for some time. Clearly the advantages we have spoken of cannot be realized in a moment, nor even in a few months. Years alone can give opportunity for the growth and development of that which is good in professional life.

Lastly, it must be progressive. Your associate comes as a pupil, he takes your directions and suggestions implicitly at first, but as time goes on and his judgment matures he will hold opinions which are now his own, worked out by his own observation and thought. Your judgment will sometimes be questioned, and you must not feel hurt or vexed; he is growing, and his place in the world will not be fulfilled till he can stand strongly and steadily alone. So gradually from pupil, assistant, associate, he comes to be your friend; possibly partner in your work, but now recognized as your equal in professional dignity, and ready to uphold the standard of excellence you have raised.

### 4. METHODS.

How can these relations between you and your associate be best realized? Three plans come to my mind. First, you may hire him, paying him a salary for his work and receiving all returns. Second, you may admit him to a share in your practice, paying him a percentage. Third, you may help to build up an independent practice for him, receiving a percentage from him.

The first plan, paying a salary, lacks the element of permanence, still more fatal is the fact that there is in it no natural growth of an independent practice for him, so there is less incentive to doing his best work.

The second plan, sharing in your practice, is that commonly used by physicians, and there has shown its value, but in dentistry, where so much depends upon the individual touch and workmanship, the division of responsibility can hardly work well in the long run, nor will a natural growth of character take place so surely.

The third plan, building up a new practice, seems upon the whole best adapted to bring out all there is good in the younger man, and make available all that is wise and helpful in the older one. This plan contemplates from the start a wholly independent practice. Each patient, even if a member of a family you are working for, becomes for the time being his, to be treated according to his best ability. It is clearly for his interest to be careful and faithful, it is also clearly yours to put all the work you can into his hands and to use your influence to fill up his time with good patients. It leads you into considerate and kindly speech, makes you careful in your criticism not to weaken the patients' confidence in him or his ability. This last plan, in which I have had long experience, is the one which I approve, for it seems to me that it possesses just the right elements to make it fit perfectly into the relations between principal and associate as we have seen them.

It will be wise, I think, to give full details of such a plan as they have been gradually worked out. It requires first a period of instruction or probation of uncertain length, followed by a period of association for a fixed time. The agreement is that the principal provides free of expense an office, furniture, attendance, instruction, criticism, advice, and patients as far as possible, while in return for this the associate agrees to pay during the period of association a definite percentage of all his work.

During the period of instruction or probation the pupil is not allowed to charge for his services at all, but a small charge may be made, to such patients as can pay, for materials and small expenses, which is paid directly to the principal. The pupil receives nothing for his work during this period, which may vary from six months to two years according to his ability and faithfulness, depending for its duration wholly upon the judgment of the principal. It is clearly to the interest of the principal to make the period as short as he can so that he may receive some adequate return for his outlay both of money and time, but he cannot properly terminate it until he is prepared to say of the young man, "He does thoroughly good work," for his own reputation is at stake.

During the period of association the principal introduces patients to the associate, gives advice, supervises work, is consulted in all difficult cases, and, in short, stands under the younger man, and allows him to build his professional life upon him. The associate makes a regular monthly return of his work, and pays a fixed percentage of what he receives after deducting expenses. After the fixed period of association, the same may be renewed at pleasure for a longer or a shorter time at the same or a lower percentage.

In presenting these details to you I am led by the fact that circumstances have made me study the practical working of such a scheme from both sides for over thirty years, and it is in view of this long experience that I speak with so much assurance. If what I have written shall be of use to any man burdened with a large and growing practice, who feels that he needs help, I shall be very thankful.

In conclusion, let me give you a bit of history. There is now living in retirement in Cambridge, Mass., an old man, blind for the past thirty years and so crippled by rheumatism as to be entirely helpless but for the devotion of a loving and faithful wife. During his years of active life he gathered round him a band of young men, and by example and precept so stimulated them to effort that the impulse of his personality still lingers among them. They have in turn endeavored to pass the torch to others, till there are now about twenty men in active practice (eight among the list of members of this Institute), besides five who have passed away. All are trying to uphold the sound principles of honest and faithful work which he taught. What a comfort to him is this thought, and what better monument can Dr. E. J. Dunning have than these lives, working out an impulse derived directly or indirectly from him! He may well say, *"Si monumentum quæris circumspice."*

